# Structural Characteristics of Facial Sagging: A Quantitative Analysis Linking Visual Assessment to Dermal, Fat, and Muscle Properties

**DOI:** 10.1111/jocd.70896

**Published:** 2026-05-14

**Authors:** Megumi Oya, Yuki Takeuchi, Yumi Touma, Kentaro Yamazaki

**Affiliations:** ^1^ HYOJO Science Lab, Research and Development Department YA‐MAN Ltd. Tokyo Japan

**Keywords:** dermis, facial sagging, muscle, regression analysis, sagging index, structural aging, subcutaneous fat

## Abstract

**Background:**

Facial sagging is associated with changes in underlying facial structures, including the dermis, subcutaneous fat, and muscle layers. However, previous studies have focused primarily on surface appearance, and quantitative evaluations linking sagging to individual internal structures remain limited, making it difficult to identify key determinants of sagging and to optimize intervention targets.

**Objective:**

This study aimed to clarify, using statistical analyses and predictive models, the contributions of quantitative structural parameters of the dermis, subcutaneous fat, and muscle to facial sagging, and to obtain fundamental insights for individualized evaluation and intervention optimization.

**Methods:**

A total of 220 women aged 20–79 years were enrolled. Facial sagging was assessed using the Merz Scale. Dermal properties were quantitatively measured. The thickness and echogenicity of subcutaneous fat and muscle layers were evaluated using high‐resolution ultrasound. Skin displacement and volume increase associated with postural changes were calculated as dynamic sagging indices. Associations between structural characteristics and sagging indices were analyzed using correlation and multiple regression analyses.

**Results:**

Sagging appearance was significantly correlated with dermal viscoelasticity, subcutaneous fat thickness, and zygomaticus major muscle thickness. Regression analysis indicated that sagging was jointly influenced by dermal, fat, and muscle characteristics, with relative structural contributions varying by facial site and age. Dynamic sagging indices were significantly correlated with age and Merz scores.

**Conclusion:**

This study demonstrated that facial sagging is not attributable to aging of a single layer but results from overlapping changes in the dermis, subcutaneous fat, and muscle layers, highlighting the necessity of integrated structural evaluation.

**Trial Registration:**

UMIN Clinical Trials Registry: UMIN000060199

## 1 Introduction

Facial sagging is a major cosmetic concern associated with aging, and its causes are thought to involve not only changes at the skin surface but also alterations in deeper facial structures, including the dermis, subcutaneous fat, and facial muscles [1, 2]. However, quantitative evaluations of individual internal structures and their relationships with changes in facial appearance remain limited.

In this study, multiple quantitative measurements of internal facial structures (dermis, subcutaneous fat, and facial muscles) were obtained, and a mathematical model was constructed to predict their associations with visually assessed facial sagging. This approach aims to quantitatively capture the factors constituting facial sagging and obtain fundamental insights for evaluation and intervention tailored to individual conditions.

## 2 Methods

### 2.1 Subjects

This cross‐sectional observational study was conducted between July and October 2025 and included Japanese women. A total of 220 participants aged 20–79 years with a body mass index (BMI) of 15.0 to < 30.0 kg/m^2^ were enrolled. The cohort consisted of 40 women in each decade from their 20s to 60s and 20 women in their 70s. Exclusion criteria included a history of cosmetic medical procedures (laser treatment, injections, or surgery), skin diseases, severe underlying diseases, and pregnancy. All participants received both oral and written explanations of the study, and written informed consent was obtained. This study was conducted in accordance with the Declaration of Helsinki and was approved by the Ethics Review Committee of Shiba Palace Clinic (approval no. 159502_rn‐40601).

### 2.2 Measurements

#### 2.2.1 Visual Assessment of Facial Sagging

To quantitatively assess facial sagging in the midface and lower face, sagging severity was evaluated using the Merz Aesthetic Scales, each scored on a 5‐point grading scale ranging from 0 (none) to 4 (severe) [3, 4, 5]. Merz scores were independently assessed by two experienced evaluators, and the mean score was used for analysis.

Sagging was evaluated in the following three regions:

- Upper cheek fullness
- Jawline
- Neck volume

#### 2.2.2 Internal Facial Structure Measurements

Each layer of facial structure was evaluated using the following equipment and methods (Figure S1).

##### 2.2.2.1 Dermis

Dermal viscoelasticity was evaluated using a suction‐based skin elasticity measurement device (Cutometer; Courage + Khazaka, Cologne, Germany) equipped with a 6‐mm probe and operated at a suction pressure of 450 mbar. Standard deformation parameters (R0–R8) were obtained at three sites on the right side of the face: the cheek, lateral canthus, and submandibular region. Four measurements were obtained at each site, and the mean value was used for subsequent analyses. The definitions of each parameter are provided in Table S1.

In addition, dermal intensity, skin thickness, and Low‐Echogenic Band (LEB) were measured using the DermaLab system (Cortex Technology, Hadsund, Denmark). Each parameter was measured five times at each site, and the mean of the three values obtained after excluding the maximum and minimum values was calculated.

##### 2.2.2.2 Subcutaneous Fat

Ultrasound imaging was performed using a SONIMAGE MX ultrasound system (Konica Minolta, Tokyo, Japan) to obtain images of the subcutaneous fat tissue at four sites on the right side of the face: the upper cheek, lower cheek, lateral cheek, and submandibular region. Both the ultrasound operator and image analyst were trained by a dermatologist. In a preliminary study, no significant differences in tissue thickness measurements between ultrasound and magnetic resonance imaging (MRI) were observed. The region corresponding to subcutaneous fat was extracted from each echo image, and subcutaneous fat thickness and subcutaneous fat echo intensity (EI) were calculated using the image analysis software WinRoof. Subcutaneous fat EI was quantified as the mean grayscale value ranging from 0 to 255.

##### 2.2.2.3 Muscle

Ultrasound imaging was used to obtain echo images of the zygomaticus major muscle (ZM) and masseter muscle (MM) on the right side of the face. Regions corresponding to each muscle were extracted from the echo images, and muscle thickness (ZMT and MMT) and muscle echo intensity (ZMEI and MMEI) were calculated using WinRoof. Muscle EI was quantified as the mean grayscale value ranging from 0 to 255.

#### 2.2.3 Assessment of Facial Morphological Changes Between Supine and Standing Positions (Figure S2)

Three‐dimensional facial images were acquired in two postures, supine and standing, using the 3D‐LifeViz Mini system (QuantifiCare, Valbonne, France). In the standing position, participants stood with their back and occiput against a wall.

The head position was standardized using anatomical landmarks, with the tragus (Trg) and subnasale (Sn) serving as reference points. The head angle was adjusted so that the Trg–Sn plane was perpendicular to the back plane in both postures.

Image analysis was performed using DermaPix software (QuantifiCare, Valbonne, France), which was used to reconstruct three‐dimensional facial geometry, set landmarks, and calculate changes between the two postures in the following parameters:

- Skin displacement distance (total, vertical, and horizontal displacement)
- Skin displacement angle
- Increase in cheek volume

Based on these measurements, dynamic sagging indices were defined to represent tissue responses to gravitational loading.

### 2.3 Measurement Conditions

Before the measurements, subjects were cleansed and allowed to rest for 20 min in a controlled environment at 21.8°C ± 0.6°C and 50.8% ± 2.3% relative humidity.

### 2.4 Statistical Analysis

Relationships between continuous variables were assessed using Spearman's correlation coefficients. To account for multiple comparisons, *p* values were adjusted using the Holm method. Group comparisons among age groups were performed using one‐way analysis of variance (ANOVA). Multiple linear regression analyses were conducted using Python (version 3.12.12) to evaluate the associations between sagging indices (dependent variables) and quantitative measurements of internal facial structures (independent variables). Multicollinearity among independent variables was assessed using the variance inflation factor (VIF). The level of statistical significance was set at *p* < 0.05 for all two‐tailed tests.

## 3 Results

### 3.1 Correlation Between Merz Scores and Age

**FIGURE 1:**
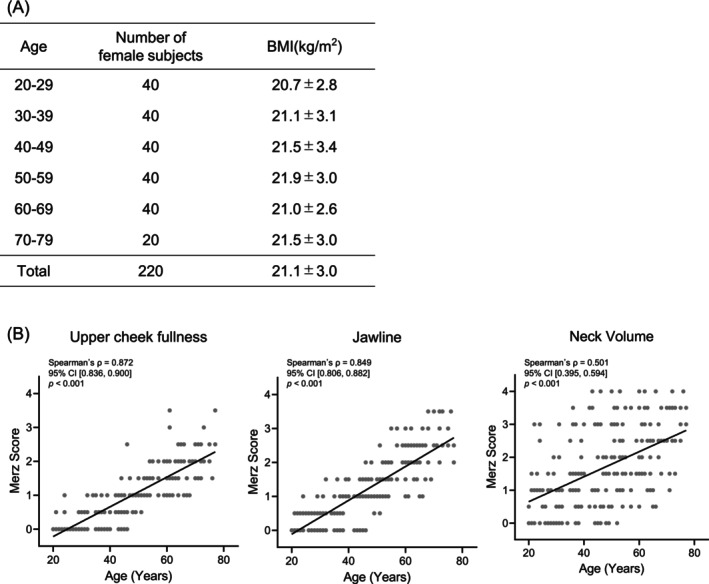
Subject characteristics. (A) Number of subjects in each age group and their mean body mass index (BMI, kg/m^2^). BMI values are presented as mean ± standard deviation. All participants were female. (B) Scatter plots showing the correlations between age (years) and three Merz scores (upper cheek fullness, jawline, and neck volume). Each panel shows individual data points (gray dots) and the regression line. Spearman's correlation coefficient (*ρ*), 95% confidence interval, and *p* value are shown in each panel. The sample size was *n* = 220.

A total of 220 Japanese women aged 20–79 years were included in the analysis. The mean BMI was 21.1 ± 3.0 kg/m^2^, and one‐way analysis of variance (ANOVA) showed no significant differences in BMI among age groups (*p* = 0.879), indicating comparable body size across groups (Figure 1A).

Significant positive correlations were observed between age and Merz scores for upper cheek fullness (*ρ* = 0.872, 95% CI [0.836, 0.900]), jawline (*ρ* = 0.849, 95% CI [0.806, 0.882]), and neck volume (*ρ* = 0.501, 95% CI [0.395, 0.594]) (*p* < 0.001 for all), indicating that Merz scores increased with age in the study population (Figure 1B).

### 3.2 Dermal Characteristics Associated With Merz Scores

**FIGURE 2:**
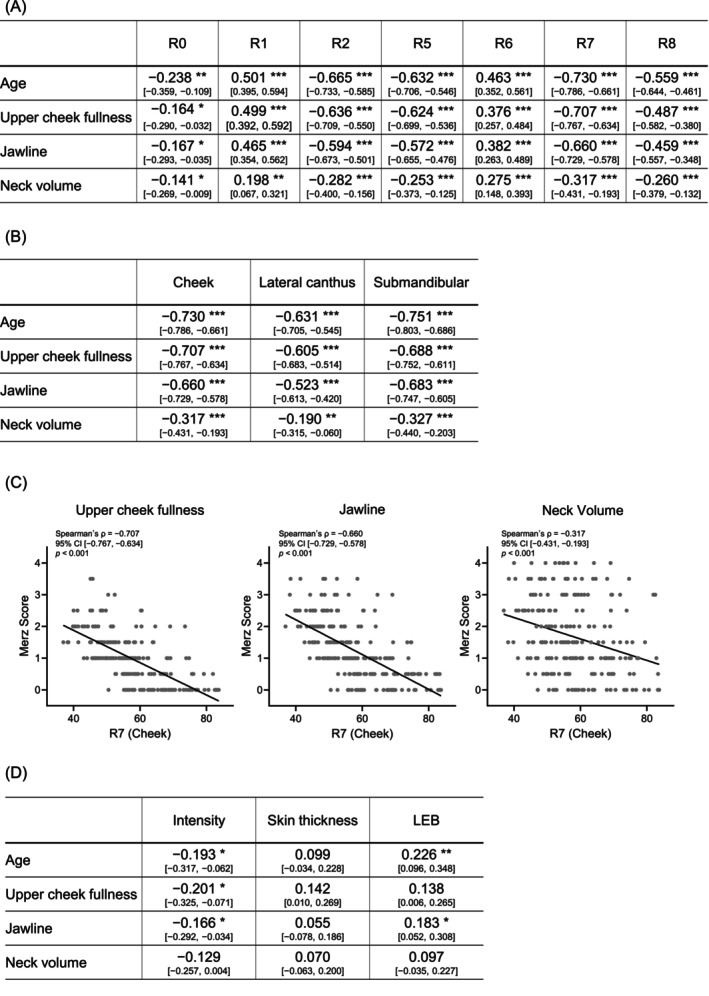
Dermal characteristics correlated with Merz scores. (A) Summary of Spearman's correlation coefficients (*ρ*) between cheek dermal viscoelastic parameters and age and the three Merz scores. Correlation coefficients and their 95% confidence intervals are shown (*n* = 220). (B) Summary of Spearman's correlation coefficients (*ρ*) between R7 measured at each facial site and age and the three Merz scores. Correlation coefficients and their 95% confidence intervals are shown (*n* = 220). Cheek indicates the cheek region; lateral canthus indicates the lateral canthal region; submandibular indicates the submandibular region. (C) Scatter plots showing the correlations between R7 and the three Merz scores (upper cheek fullness, jawline, and neck volume). Each panel shows individual data points (gray dots) and the regression line. Spearman's correlation coefficient (*ρ*), 95% confidence interval, and *p* value are shown in each panel (*n* = 220). (D) Summary of Spearman's correlation coefficients (*ρ*) between cheek dermal intensity, skin thickness, and LEB and age and the three Merz scores. Correlation coefficients and their 95% confidence intervals are shown (*n* = 220). *p < 0.05; **p < 0.01; ***p < 0.001. Values without asterisks are not statistically significant.

To examine the association between facial sagging appearance and dermal characteristics, dermal viscoelasticity was measured at three facial sites—the cheek, lateral canthus, and submandibular region—using a Cutometer with a 6‐mm probe. Correlation coefficients between age and dermal viscoelastic parameters at the cheek for each Merz score are shown in Figure 2A. Age was significantly negatively correlated with the viscoelastic parameters R2, R5, R7, and R8 (*p* < 0.001), and these parameters also showed significant negative correlations with all three Merz scores (upper cheek fullness, jawline, and neck volume) (*p* < 0.001). In contrast, R1 and R6 were positively correlated with both age and each Merz score (*p* < 0.01). R0 was correlated with age, upper cheek fullness, and jawline, and no correlation was observed with neck volume. Among the viscoelastic parameters, R7 showed the strongest correlations with each Merz score. Therefore, correlations between R7 and the Merz scores were further examined at the cheek, lateral canthus, and submandibular region (Figure 2B). Significant correlations were observed at all sites (*p* < 0.001), with the correlation being particularly strong at the cheek. Dermal intensity, skin thickness, and LEB at the cheek were subsequently assessed using the DermaLab (Figure 2D). Intensity showed significant correlations with age and with the Merz scores for upper cheek fullness and jawline, and LEB was correlated with age or with the Merz scores for jawline. In contrast, skin thickness was not correlated with either of these variables (*p* > 0.05).

Comprehensive Spearman correlation analyses between age, the three Merz scores, and each measured variable are provided in Table S2.

### 3.3 Subcutaneous Fat Tissue Characteristics Associated With Merz Scores

To evaluate the association between facial sagging appearance and subcutaneous fat characteristics, subcutaneous fat thickness and EI were assessed at four facial sites—the upper cheek, lower cheek, lateral cheek, and submandibular region—using ultrasound imaging and image analysis.

**FIGURE 3:**
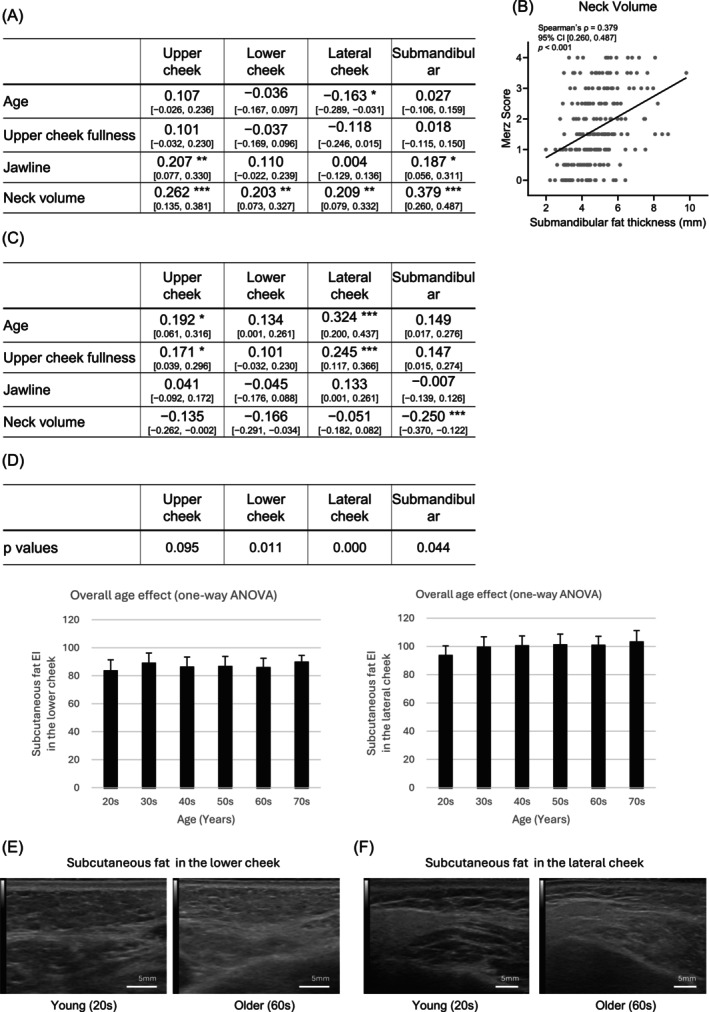
Subcutaneous fat characteristics correlated with Merz scores. (A) Summary of Spearman's correlation coefficients (*ρ*) between subcutaneous fat thickness and age and the three Merz scores. Correlation coefficients and their 95% confidence intervals are shown (*n* = 220). Upper cheek, lateral cheek, lower cheek, and submandibular indicate the corresponding measurement regions. (B) Scatter plot showing the correlation between the Merz score for neck volume and submandibular fat thickness. The panel shows individual data points (gray dots) and the regression line. Spearman's correlation coefficient (*ρ*), 95% confidence interval, and *p* value are shown in the panel (*n* = 220). (C) Summary of Spearman's correlation coefficients (*ρ*) between subcutaneous fat EI in the upper cheek, lower cheek, lateral cheek, and submandibular regions and age and the three Merz scores. Correlation coefficients and their 95% confidence intervals are shown (*n* = 220). (D) The *p* values represent overall age effects assessed by one‐way ANOVA for each facial region. Mean ± SD values of subcutaneous fat EI measured in the lower cheek and lateral cheek regions across age groups are shown (*n* = 220). (E) Representative ultrasound images of subcutaneous fat in the lower cheek from young and older individuals. (F) Representative ultrasound images of subcutaneous fat in the lateral cheek from young and older individuals. *p < 0.05; **p < 0.01; ***p < 0.001. Values without asterisks are not statistically significant.

Correlation coefficients between subcutaneous fat thickness, age, and Merz scores are shown in Figure 3A. Age showed a weak negative correlation only with fat thickness in the lateral cheek (*ρ* = −0.163, 95% CI [−0.289, −0.031], *p* < 0.05), while no significant correlations were observed at the other sites (*p* > 0.05). Fat thickness in the upper cheek and submandibular region showed significant correlations with the Merz scores for jawline and neck volume, whereas fat thickness in the lower cheek and lateral cheek showed significant correlations only with the Merz scores for neck volume (*p* < 0.05). Notably, the Merz score for neck volume showed significant positive correlations with fat thickness at all measured sites (*p* < 0.05). A representative scatter plot showing the relationship between the Merz score for neck volume and submandibular fat thickness is presented in Figure 3B (*ρ* = 0.379, 95% CI [0.260, 0.487], *p* < 0.001).

Correlation coefficients between the subcutaneous fat EI, age, and Merz scores are shown in Figure 3C. Subcutaneous fat EI increased significantly with age at all measurement sites (*p* < 0.05). With respect to Merz scores, subcutaneous fat EI in the submandibular region was significantly correlated with the Merz scores for neck volume; in the upper and lateral cheek, it was correlated with the Merz score for upper cheek fullness (*p* < 0.05).

One‐way analysis of variance across age groups revealed that subcutaneous fat EI in the lower cheek, lateral cheek, and submandibular region increased with age, whereas no significant age‐related differences were observed in the upper cheek, although a trend toward increased EI was noted (*p* = 0.095; Figure 3D). Representative ultrasound images are shown in Figure 3E,F.

Comprehensive Spearman correlation analyses between age, the three Merz scores, and each measured variable are provided in Table S2.

### 3.4 Facial Muscle Characteristics Associated With Merz Scores

To evaluate the association between facial sagging appearance and muscle characteristics, muscle thickness and EI of the zygomaticus major and masseter muscles were assessed using ultrasound imaging and image analysis.

**FIGURE 4:**
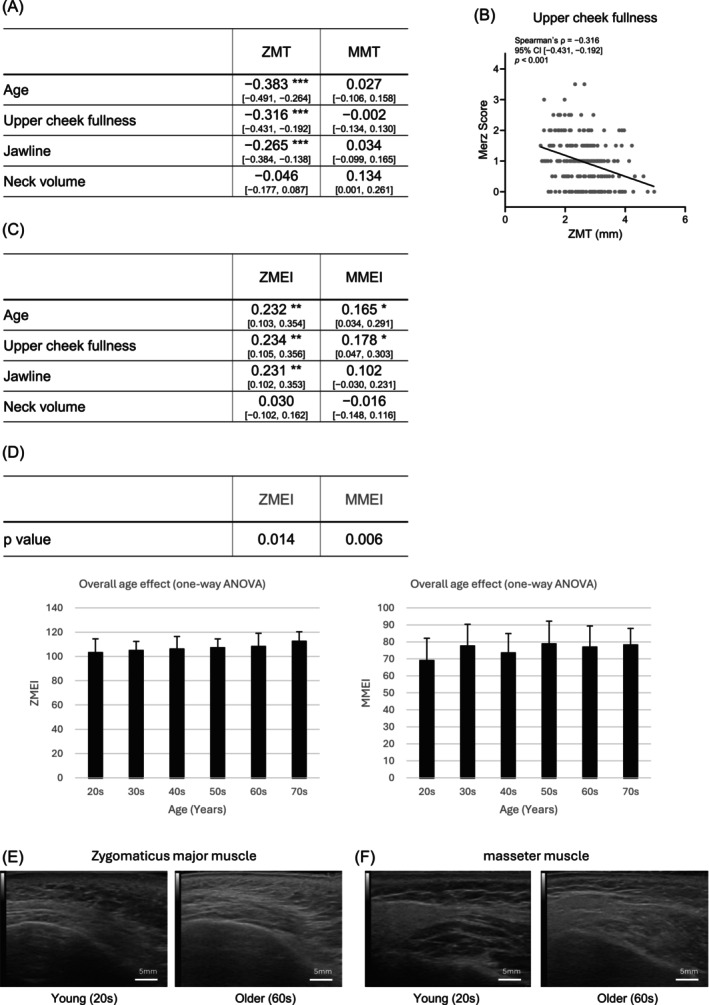
Muscle characteristics correlated with Merz scores. (A) Summary of Spearman's correlation coefficients (*ρ*) between zygomaticus major thickness (ZMT) and masseter muscle thickness (MMT) and age and the three Merz scores. Correlation coefficients and their 95% confidence intervals are shown (*n* = 220). (B) Scatter plot showing the correlation between the Merz score for upper cheek fullness and ZMT. The panel shows individual data points (gray dots) and the regression line. Spearman's correlation coefficient (*ρ*), 95% confidence interval, and *p* value are shown in the panel (*n* = 220). (C) Summary of Spearman's correlation coefficients (*ρ*) between zygomaticus major echo intensity (ZMEI) and masseter muscle echo intensity (MMEI) and age and the three Merz scores. Correlation coefficients and their 95% confidence intervals are shown (*n* = 220). (D) The *p* values represent overall age effects assessed by one‐way ANOVA for each muscle. Mean ± SD values of ZMEI and MMEI across age groups are shown (*n* = 220). (E) Representative ultrasound images of the zygomaticus major muscle in young and older individuals. (F) Representative ultrasound images of the masseter muscle in young and older individuals. *p < 0.05; **p < 0.01; ***p < 0.001. Values without asterisks are not statistically significant.

Correlation coefficients between muscle thickness, age, and Merz scores are shown in Figure 4A. Age was significantly negatively correlated with zygomaticus major muscle thickness (ZMT) (*p* < 0.001), whereas no significant correlation was observed for masseter muscle thickness (MMT). ZMT was also negatively correlated with the Merz scores for upper cheek fullness and jawline (*p* < 0.001). In contrast, MMT was not correlated with any of the Merz scores (*p* > 0.05). A representative scatter plot illustrating the relationship between the Merz score for upper cheek fullness and ZMT is shown in Figure 4B (*ρ* = −0.316, 95% CI [−0.431, −0.192], *p* < 0.001).

Regarding muscle EI, EI increased significantly with age in both the zygomaticus major and masseter muscles (Figure 4C). Zygomaticus major muscle EI (ZMEI) was significantly correlated with the Merz scores for upper cheek fullness and jawline, whereas masseter muscle EI (MMEI) was significantly correlated only with the Merz score for upper cheek fullness (*p* < 0.01). One‐way analysis of variance across age groups revealed that EI of both the zygomaticus major and masseter muscles increased significantly with age (*p* < 0.05; Figure 4D). Representative ultrasound images are shown in Figure 4E,F.

Comprehensive Spearman correlation analyses between age, the three Merz scores, and each measured variable are provided in Table S2.

### 3.5 Relationship Between Dynamic Sagging Indices and Structural Measurements

The Merz Scale evaluates facial sagging appearance using an ordinal five‐point scale; however, in this study, sagging was quantified as a continuous variable using dynamic sagging indices derived from facial morphological changes induced by changes in gravitational loading direction from the supine to standing position. Three‐dimensional facial images in both positions were acquired using the 3D‐LifeViz Mini, and the differences were used to calculate dynamic sagging indices reflecting changes in gravitational load direction.

**FIGURE 5:**
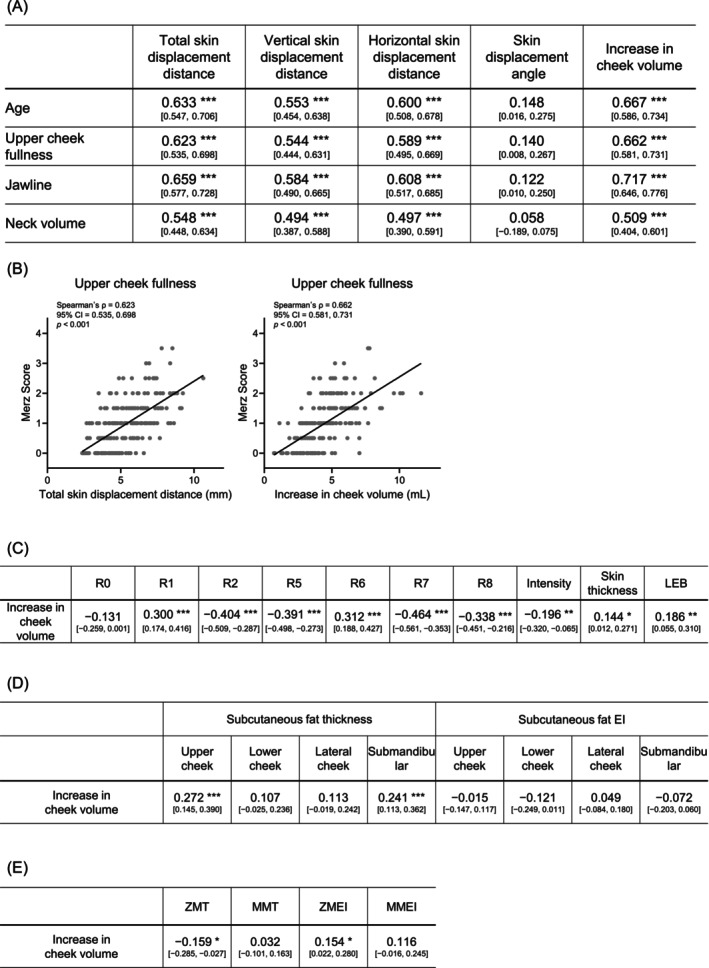
Dynamic sagging indices. (A) Summary of Spearman's correlation coefficients (*ρ*) between dynamic sagging indices and age and the three Merz scores. Correlation coefficients and their 95% confidence intervals are shown (*n* = 220). (B) Scatter plots showing the correlations between the Merz score for upper cheek fullness and two dynamic sagging indices (total skin displacement distance and increase in cheek volume). Each panel shows individual data points (gray dots) and the regression line. Spearman's correlation coefficient (*ρ*), 95% confidence interval, and *p* value are shown in each panel (*n* = 220). (C) Summary of Spearman's correlation coefficients (*ρ*) between dermal characteristics and the dynamic sagging index (increase in cheek volume). Correlation coefficients and their 95% confidence intervals are shown (*n* = 220). (D) Summary of Spearman's correlation coefficients (*ρ*) between subcutaneous fat characteristics and the dynamic sagging index (increase in cheek volume). Correlation coefficients and their 95% confidence intervals are shown (*n* = 220). (E) Summary of Spearman's correlation coefficients (*ρ*) between muscle characteristics and the dynamic sagging index (increase in cheek volume). Correlation coefficients and their 95% confidence intervals are shown (*n* = 220). *p < 0.05; **p < 0.01; ***p < 0.001. Values without asterisks are not statistically significant.

First, correlations between the dynamic sagging indices, age, and the three Merz scores were examined. Five dynamic sagging indices were calculated: total skin displacement distance, vertical skin displacement distance, horizontal skin displacement distance, skin displacement angle, and increase in cheek volume (Figure 5A). Total skin displacement distance during the transition from supine to standing showed significant positive correlations with age and all three Merz scores (*p* < 0.001). Greater sagging appearance was associated with increased skin displacement in both horizontal and vertical directions. Increase in cheek volume also showed significant correlations with age and all three Merz scores (*p* < 0.001). In contrast, the skin displacement angle did not show strong correlations with age or Merz scores. Representative scatter plots of the two indices showing significant correlations with the Merz score for upper cheek fullness are shown in Figure 5B.

Next, among the dynamic sagging indices that correlated with both age and Merz scores, the increase in cheek volume, which exhibited a strong correlation, was further analyzed in relation to quantitative measurements of the dermis, subcutaneous fat, and facial muscles (Figure 5C–E). Increase in cheek volume showed significant correlations with dermal viscoelastic parameters, particularly R7, subcutaneous fat thickness in the upper cheek and submandibular region, and ZMT and ZMEI (*p* < 0.05).

Comprehensive Spearman correlation analyses between the dynamic sagging index (increase in cheek volume) and each measured variable are provided in Table S3.

### 3.6 Contribution of Each Layer to Sagging

To evaluate the contribution of individual tissue characteristics to facial sagging, multiple linear regression models were constructed to predict each sagging index based on internal structural parameters in 220 participants. All variables were standardized prior to analysis, and multicollinearity among candidate explanatory variables was assessed using the variance inflation factor (VIF). If multicollinearity was detected (VIF ≥ 5), only the parameter showing the strongest correlation with the corresponding Merz score was retained. The contribution rates were calculated as the ratio of the absolute value of the standardized coefficient (*β*) for each independent variable obtained from the multiple regression analysis to the sum of the absolute values of all standardized coefficients. This approach allowed characteristic parameters with different units to be compared on a common scale and enabled visualization of their relative importance with respect to sagging.

After examining the goodness of fit of the regression models for each region (Table 1), particularly high explanatory power was observed for the upper cheek (adjusted *R* ^2^ = 0.486) and the jawline (adjusted *R* ^2^ = 0.461).

**TABLE 1:** Summary of multiple regression model performance.

Target	Adjusted *R* ^2^	Interpretation
Upper cheek fullness	0.486	Relatively high explanatory power
Jawline	0.461	Relatively high explanatory power
Neck volume	0.239	Moderate explanatory power
Increase in cheek volume (Young)	0.107	Low explanatory power
Increase in cheek volume (Older)	0.285	Moderate explanatory power

**FIGURE 6:**
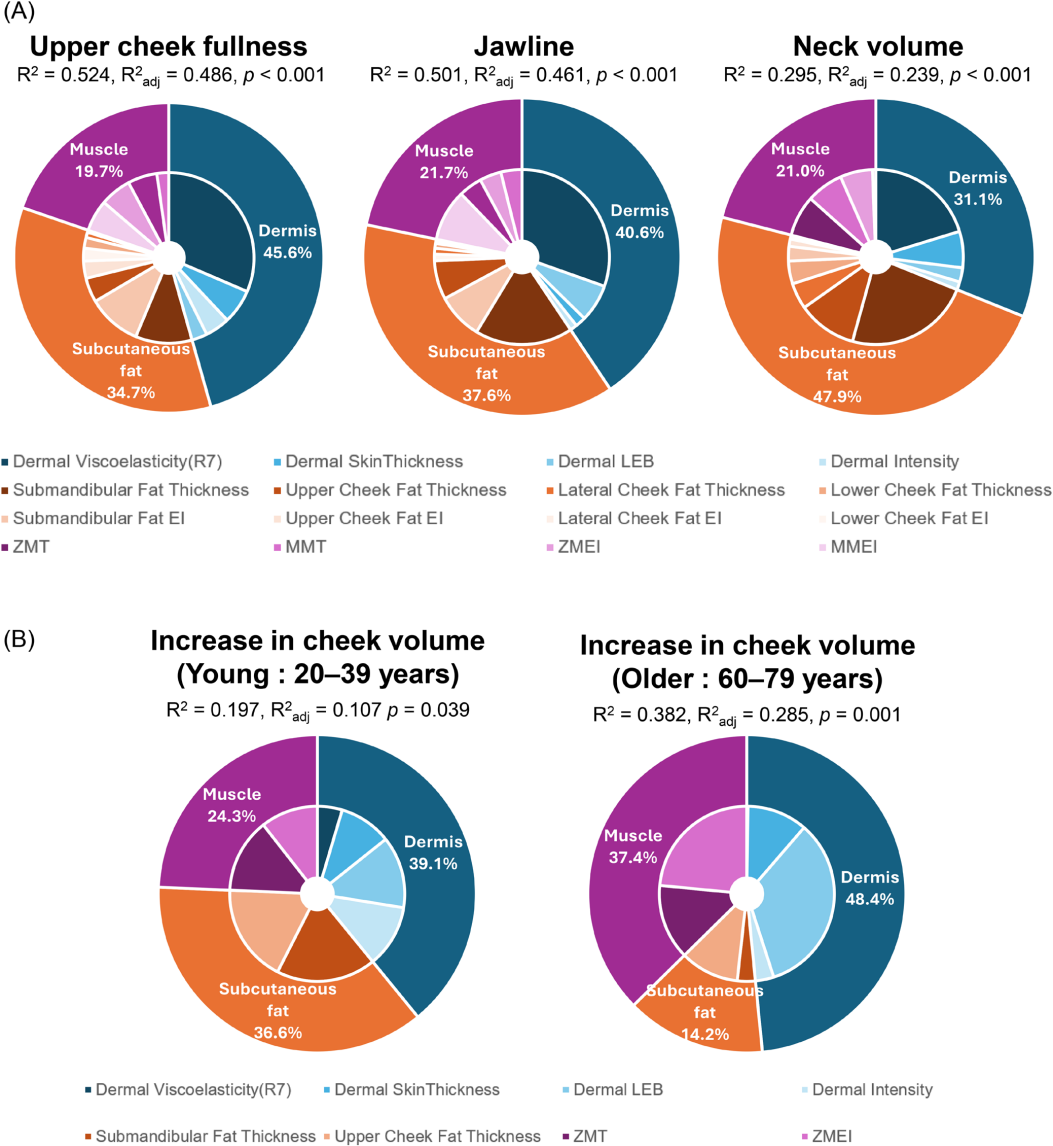
Layer‐specific contributions of structural characteristics to facial sagging indices. (A) Donut charts showing the relative contributions of dermal, subcutaneous fat, and muscle parameters to the regression models predicting upper cheek fullness, jawline, and neck volume. The outer rings represent the summed contributions of each structural layer (dermis, subcutaneous fat, and muscle), whereas the inner rings indicate the contributions of individual parameters within each layer. Model performance is shown above each chart (*R* ^2^, adjusted *R* ^2^, and *p* value). (B) Donut charts showing age‐dependent differences in the contributions of dermal, subcutaneous fat, and muscle parameters to the regression models predicting increase in cheek volume in the young (20–39 years) and older (60–79 years) groups. As in panel (A), the outer rings indicate layer‐level contributions and the inner rings indicate the contributions of individual parameters. Model performance statistics are shown above each chart (*R* ^2^, adjusted *R* ^2^, and *p* value). A Chow test revealed a significant difference between the regression models for the young (20–39 years) and older (60–79 years) groups (*F* = 18.18, *p* = 2.15 × 10^−6^).

The explanatory variables predicting each Merz score consisted of dermal characteristics, subcutaneous fat characteristics, and facial muscle characteristics. Comparing the three Merz scores, it was shown that the contribution of dermal characteristics is large in the upper cheek, and the contribution of fat characteristics is large in the submandibular region, indicating that the contribution of each layer to sagging varies by area (Figure 6A).

Next, to investigate whether the contribution of each characteristic to sagging changes with aging, linear regression models for increase in cheek volume, a sagging index expressed as a continuous quantity, were constructed for young (20–39 years) and older (60–79 years) groups, and the contribution of each item to predicting increase in cheek volume was examined. To avoid overfitting, the explanatory variables included parameters of the skin, subcutaneous fat, and facial muscles that showed significant correlations with increase in cheek volume (*p* < 0.05). The adjusted *R* ^2^ values for the young (20–39 years) and older (60–79 years) groups were 0.107 and 0.285, respectively (Table 1). Compared to the young prediction model, the older prediction model showed a decrease in the contribution of fat characteristics and an increase in the contribution of dermal and muscle characteristics (Figure 6B). To evaluate the structural difference between the regression models of the young and older groups, a Chow test was performed. The test yielded an *F*‐statistic of 18.18 and a *p* value of 2.15 × 10^−6^, indicating a statistically significant difference in the regression coefficients between the two age groups.

The relative contributions of dermal, subcutaneous fat, and muscle characteristics to facial sagging, as determined by multiple regression analysis, are provided in Table S4.

## 4 Discussion

In this study, facial sagging was first evaluated using a visual scoring system, followed by the analysis of multifaceted quantitative data describing internal facial structures. This study is characterized by a clinically oriented approach that begins with visually perceived sagging and subsequently explores its underlying structural determinants. While previous studies have primarily classified facial sagging based on quantitative measurements alone [6], the present study adopts a reverse strategy by investigating the structural basis of sagging as perceived in appearance, providing both methodological novelty and clinical relevance.

The three Merz scores—upper cheek fullness, jawline, and neck volume—increased with age in the present cohort (Figure 1), indicating that the study population exhibited typical age‐related facial sagging. We first examined the associations between dermal quantitative parameters and Merz scores. While some dermal parameters related to collagen density and thickness showed statistically significant correlations with sagging, their correlation coefficients were relatively low, suggesting a limited contribution of these static quantitative dermal parameters to the appearance of sagging. In contrast, dermal viscoelastic parameters measured using the Cutometer showed strong and consistent correlations with age and all three Merz scores, particularly those centered on R7 (Figure 2A). Taken together, these findings suggest that facial sagging appearance is not simply driven by a quantitative loss of dermal components, but rather reflects qualitative changes in the dermis, such as reduced mechanical support and recovery behavior under load, as represented by decreased dermal viscoelasticity.

Next, we examined the associations between subcutaneous fat characteristics and Merz scores, focusing on fat thickness as a quantitative parameter and echo intensity (EI) as a qualitative parameter. Fat thickness showed significant correlations with Merz scores at multiple facial sites, with a consistent association observed for the neck volume score across all measured regions (Figure 3A). In contrast, upper cheek fullness did not correlate with fat thickness at any site, suggesting that the volume of subcutaneous fat primarily contributes to sagging in the lower face rather than the midface.

Subcutaneous fat EI was also correlated with several Merz scores (Figure 3C). Increased EI has been reported to be associated with enhanced fibrosis and increased stromal components in adipose tissue [7, 8]. Fibrotic changes in adipose tissue can lead to excessive accumulation of extracellular matrix components, such as collagen, and tissue stiffening, thereby altering the mechanical properties of the fat layer [9, 10, 11]. These changes are thought to increase tissue stiffness and reduce mechanical flexibility of the fat layer. Although most evidence regarding adipose tissue fibrosis originates from obesity‐related fat depots, similar structural alterations may occur in facial subcutaneous fat and influence its mechanical properties. The present findings suggest that such qualitative changes in subcutaneous fat may contribute to alterations in facial sagging appearance. When subcutaneous fat characteristics were examined in relation to age, fat thickness showed no significant correlation with age, whereas fat EI increased significantly with aging. Consistently, analysis of variance across age decades demonstrated a significant age‐related increase in fat EI (Figure 3D). These results indicate that fat EI can serve as a continuous quantitative marker of age‐related qualitative changes in subcutaneous fat.

These findings indicate that qualitative changes in subcutaneous fat, reflected by increased subcutaneous fat EI, contribute to the appearance of facial sagging. In contrast, lower‐face sagging was strongly influenced by subcutaneous fat thickness, which was independent of age, suggesting that the sagging phenotype is shaped by the combined effects of both qualitative and quantitative subcutaneous fat characteristics.

Regarding the muscle layer, ZMT decreased with age and showed a significant negative correlation with sagging in the midface and lower face (Figure 4A). The zygomaticus major muscle plays a significant role in elevating and supporting the midfacial soft tissue, and its age‐related thinning is therefore likely to enhance cheek sagging through reduced structural support of the overlying skin and subcutaneous fat. This interpretation is consistent with previous findings suggesting that mimetic muscles play a role in maintaining facial soft tissue position [2]. In contrast, the masseter is a deep muscle primarily responsible for mastication, and its direct contribution to skin support may be limited. From an aging perspective, ZMT showed a significant negative correlation with age, whereas MMT did not. Although skeletal muscle generally undergoes age‐related atrophy, the masseter is continuously exposed to functional loading during mastication, and its thickness is known to be influenced by factors such as tooth loss, bruxism, and clenching habits in addition to aging [12, 13]. These findings suggest that site‐specific functional demands may strongly modulate muscle thickness, making it difficult to directly link muscle quantity alone to sagging severity.

In contrast, muscle EI showed a significant age‐related increase, similar to that observed for subcutaneous fat, and correlated with multiple Merz scores (Figure 4C,D). Increased muscle echo intensity is known to reflect intramuscular fat infiltration and, to some extent, fibrotic changes, indicating qualitative deterioration of muscle tissue [14, 15]. These results suggest that age‐related changes in muscle quality, rather than muscle thickness alone, may reduce the ability of the muscle to support overlying soft tissues, thereby contributing to the appearance of facial sagging.

One of the key findings of this study was that the dynamic sagging indices derived from supine‐to‐standing positional changes showed strong correlations with both age and all three Merz scores. In particular, the increase in cheek volume demonstrated a robust correlation with Merz scores and was also significantly correlated with structural characteristics of the dermal, subcutaneous fat, and muscle layers (Figure 5A,C–E).

The Merz Scale is a clinically practical tool for evaluating facial sagging; however, because it is an ordinal, five‐point scale based on visual assessment, it has inherent limitations in detecting subtle changes and is subject to inter‐examiner variability. In contrast, conventional assessments of sagging have largely relied on static facial morphology, whereas the dynamic sagging index characterizes the tissue response to changes in gravitational loading. Although further validation is required, by quantifying sagging as a continuous variable with improved objectivity and reproducibility, the dynamic sagging index may provide a valuable tool for monitoring age‐related changes and for objectively evaluating the effects of clinical or cosmetic interventions.

Multiple regression analysis demonstrated that each Merz score and the dynamic sagging index are explained by a combination of structural factors across the dermal, subcutaneous fat, and muscle layers. Importantly, the relative contribution of these components varied by facial site, with dermal characteristics contributing most prominently to upper cheek sagging, whereas subcutaneous fat characteristics contributed to a greater extent to submental sagging (Figure 6A). In addition, the contribution of each structural characteristic to sagging differed across age groups (Figure 6B). In younger individuals, dermal, fat, and muscle characteristics contributed relatively evenly, whereas in older individuals, the relative contributions of dermal and muscle characteristics increased while that of subcutaneous fat decreased. These findings indicate that facial sagging may arise from the combined effects of multiple structural changes rather than a single dominant factor, highlighting the importance of a multi‐layered evaluation. Quantifying the contribution of each structural layer may therefore provide a basis for estimating individual sagging tendencies from objective measurements and for developing personalized evaluation and intervention strategies tailored to structural characteristics and stages of aging.

The framework proposed in this study is highly practical, as it directly links the visual appearance of sagging with quantitative information on internal facial structure. This structure‐based approach offers a new perspective for understanding facial sagging and may be extended in future studies to develop more advanced sagging prediction algorithms and structure‐specific, individualized treatment evaluation indices.

### 4.1 Limitations

This study has several limitations. First, its cross‐sectional design precludes the evaluation of temporal changes, such as longitudinal age‐related progression or the effects of interventions. Future studies incorporating longitudinal follow‐up and pre–post intervention designs will be necessary to clarify causal relationships and to assess the reversibility of the proposed sagging‐related indicators.

Second, the present analysis was limited to Japanese women, and potential differences related to sex or ethnicity were not examined. Expanding the study population to include males and individuals from diverse ethnic backgrounds will be important to evaluate the generalizability of the structural contribution model proposed in this study.

## 5 Conclusion

In this study, we quantitatively assessed facial sagging based on visual appearance and investigated its relationship with the underlying dermal, subcutaneous fat, and muscle characteristics. Our findings demonstrated significant associations between sagging and the mechanical properties of the dermis, as well as the quantitative properties of the subcutaneous fat and muscle layers. This suggests that facial sagging is not caused by a single factor but rather results from the complex interaction of multiple structural components. In addition, the contribution of each structural element to sagging varies depending on the region and age, highlighting the need for comprehensive assessment. Furthermore, the observed increase in subcutaneous fat and muscle EI with aging suggests that these parameters may serve as indicators of qualitative aging of the fat and muscle layers.

Moreover, the dynamic sagging indices derived from supine‐to‐standing postural changes enabled continuous quantification of tissue responses to gravitational loading while maintaining consistency with conventional visual assessment scales. These indices may therefore complement static evaluations by providing an objective and reproducible approach to sagging assessment.

In conclusion, this study proposes a framework for decomposing facial sagging into structural components—dermis, subcutaneous fat, and muscle—providing a basis for layer‐oriented evaluation and intervention strategies tailored to individual structural characteristics.

## Author Contributions

All authors contributed to the study design, data analysis and interpretation, and manuscript review. Megumi Oya, Yuki Takeuchi, and Yumi Touma collected the data; Megumi Oya, Yuki Takeuchi, and Kentaro Yamazaki performed the statistical analysis; Megumi Oya wrote the manuscript; Kentaro Yamazaki served as the principal investigator and supervised the study design, execution, and ethical compliance. All authors approved the final manuscript.

## Funding

This work was supported by YA‐MAN Ltd.

## Conflicts of Interest

All authors are employees of YA‐MAN Ltd.

## Supporting information


**Figure S1:** Measurement locations. (A) Measurement locations for dermal characteristics: (1) cheek, (2) lateral canthus, and (3) submandibular region. (B) Measurement locations for subcutaneous fat characteristics: (1) upper cheek, (2) lower cheek, (3) lateral cheek, and (4) submandibular region. Representative ultrasound images are shown. In the ultrasound images, regions outlined in orange were defined as subcutaneous fat tissue and used for image analysis. (C) Measurement locations for muscle characteristics: (1) zygomaticus major and (2) masseter muscle. Representative ultrasound images are shown. In the ultrasound images, regions outlined in orange were defined as muscle tissue and used for image analysis.
**Figure S2:** Facial morphological changes induced by changes in gravitational loading direction. (A) Schematic diagram illustrating the acquisition of three‐dimensional facial data in supine and standing positions. Trg, tragus; Sn, subnasale. (B) Skin displacement induced by changes in gravitational loading direction between supine and standing positions in young and older individuals. Arrows indicate the distance and direction of skin displacement from the supine to standing position. (C) Increase in cheek volume induced by changes in gravitational loading direction between supine and standing positions in young and older individuals. The color scale indicates the magnitude of volume increase from the supine to standing position.

**Table S1:** Definitions of dermal viscoelastic parameters measured by cutometer.
**Table S2:** Comprehensive correlation analysis between age, Merz scores, and structural variables. Comprehensive Spearman correlation analysis between age, the three Merz scores, and each measured variable. The table presents Spearman's correlation coefficient (*ρ*), 95% confidence intervals (CI), original *p* values, and Holm–Bonferroni corrected *p* values. Statistical significance was determined based on the Holm‐adjusted *p* values.
**Table S3:** Comprehensive correlation analysis between dynamic sagging index and structural variables. Comprehensive Spearman correlation analysis between the dynamic sagging index (increase in cheek volume) and each measured variable. The table includes Spearman's correlation coefficient (*ρ*), 95% confidence intervals (CI), and *p* values.
**Table S4:** Relative contribution of dermal, subcutaneous fat, and muscle characteristics to facial sagging estimated by multiple regression analysis. (A) Summary of the relative contributions of dermal, subcutaneous fat, and muscle characteristics to the three Merz scores. (B) Summary of the relative contributions of dermal, subcutaneous fat, and muscle characteristics to the dynamic sagging index (increase in cheek volume).

## Data Availability

The data that support the findings of this study are available on request from the corresponding author. The data are not publicly available due to privacy or ethical restrictions.
