# Supplementary material for: Structural Characteristics of Facial Sagging: A Quantitative Analysis Linking Visual Assessment to Dermal, Fat, and Muscle Properties
Source: J Cosmet Dermatol. 2026 May 14;25:e70896. doi: 10.1111/jocd.70896 (PMC13176704; doi:10.1111/jocd.70896)
Supplement: Supplementary file 1 — Figure S1: Measurement locations. (A) Measurement locations for dermal characteristics: (1) cheek, (2) lateral canthus, and (3) submandibular region. (B) Measurement locations for subcutaneous fat characteristics: (1) upper cheek, (2) lower cheek, (3) lateral cheek, and (4) submandibular region. Representative ultrasound images are shown. In the ultrasound images, regions outlined in orange were defined as subcutaneous fat tissue and used for image analysis. (C) Measurement locations for muscle characteristics: (1) zygomaticus major and (2) masseter muscle. Representative ultrasound images are shown. In the ultrasound images, regions outlined in orange were defined as muscle tissue and used for image analysis. Figure S2: Facial morphological changes induced by changes in gravitational loading direction. (A) Schematic diagram illustrating the acquisition of three‐dimensional facial data in supine and standing positions. Trg, tragus; Sn, subnasale. (B) Skin displacement induced by changes in gravitational loading direction between supine and standing positions in young and older individuals. Arrows indicate the distance and direction of skin displacement from the supine to standing position. (C) Increase in cheek volume induced by changes in gravitational loading direction between supine and standing positions in young and older individuals. The color scale indicates the magnitude of volume increase from the supine to standing position. [file JOCD-25-e70896-s001.pptx]

## Slide 1
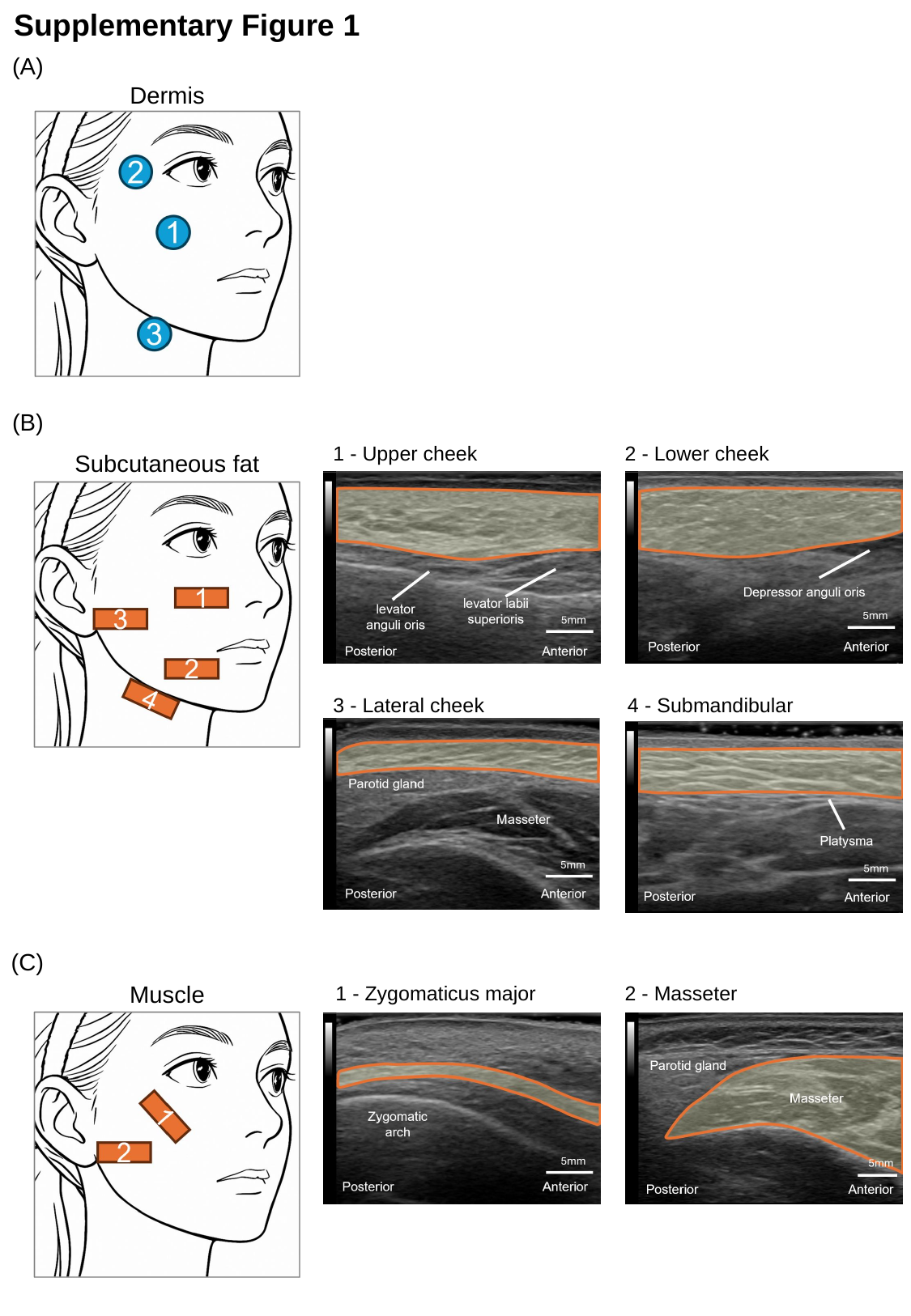

Supplementary Figure 1
(A)
Dermis
(B)
1 - Upper cheek
2 - Lower cheek
Subcutaneous fat
3 - Lateral cheek
4 - Submandibular
(C)
Muscle
1 - Zygomaticus major
2 - Masseter

## Slide 2
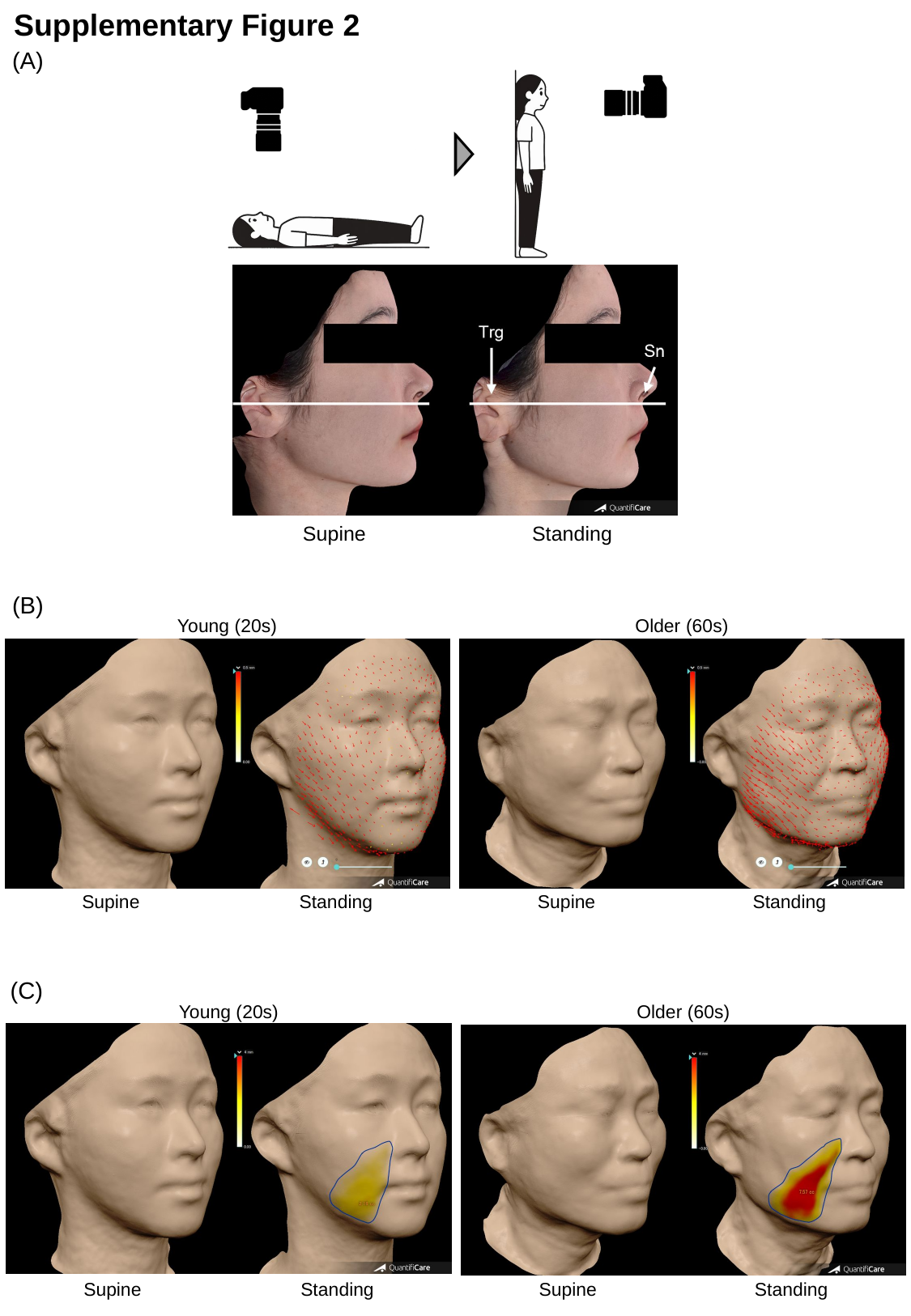

Supplementary Figure 2
(A)
Supine 　　　　　 Standing
(B)
Young (20s)
Older (60s)
Supine 　 　　　　Standing
Supine 　　　　　 Standing
(C)
Young (20s)
Older (60s)
Supine 　　　　　 Standing
Supine 　　　　　 Standing
